# Cemented versus Uncemented Hemiarthroplasty for Femoral Neck Fractures in Elderly Patients: A Meta-Analysis

**DOI:** 10.1371/journal.pone.0068903

**Published:** 2013-07-23

**Authors:** Tao Li, Qianyu Zhuang, Xisheng Weng, Lei Zhou, Yanyan Bian

**Affiliations:** Department of Orthopaedic Surgery, Peking Union Medical College Hospital, Peking Union Medical College, Chinese Academy of Medical Science, Beijing, China; Marienhospital Herne - University of Bochum, Germany

## Abstract

**Objective:**

Controversy still exists regarding using cemented or uncemented hemiarthroplasty for femoral neck fractures in elderly patients. The aim of this study is to compare the effectiveness and safety of the two surgical techniques in femoral neck fracture patients over 70 years old.

**Methods:**

We searched PUBMED, EMBASE, Cochrane Library, CNKI and VIP Database from inception to December 2012 for relevant randomized controlled trials (RCTs). Outcomes of interest include postoperative hip function, residue pain, complication rates, mortality, reoperation rate, operation time and intraoperative blood loss. Odds ratios (OR) and weighted mean differences (WMD) from each trial were pooled using random-effects model or fixed-effects model given on the heterogeneity of the included studies.

**Results:**

7 RCTs involving 1,125 patients (1,125 hips) were eligible for meta-analysis. Our results demonstrate that cemented hemiarthroplasty is associated with better postoperative hip function (OR = 0.48, 95% CI, 0.31–0.76; P = 0.002), lower residual pain (OR = 0.43, 95%CI, 0.29–0.64; P<0.0001), less implant-related complications (OR = 0.15, 95%CI, 0.09–0.26; P<0.00001) and longer operation time (WMD = 7.43 min, 95% CI, 5.37–9.49 min; P<0.00001). No significant difference was observed between the two groups in mortality, cardiovascular and cerebrovascular complications, local complications, general complications, reoperation rate and intraoperative blood loss.

**Conclusions:**

Compared with uncemented hemiarthroplasty, the existing evidence indicates that cemented hemiarthroplasty can achieve better hip function, lower residual pain and less implant-related complications with no increased risk of mortality, cardiovascular and cerebrovascular complications, general complications, local complications and reoperation rate in treating elderly patients with femoral neck fractures.

## Introduction

Femoral neck fracture is a most common injury which can lead to increased postoperative morbidity and mortality in senior patients. Hemiarthroplasty, as an effective treatment [1]–[2], contributes to early ambulation and good functional recovery. However, there has been persistent controversy over whether cemented hemiarthroplasty (CH) or uncemented hemiarthroplasty (UCH) is preferable for the patient population. CH may bring low periprosthetic fractures and prosthetic loosening whereas it may lead to embolism and decreased cardiac output with the insertion of bone cement [3]–[6]. However, there is a higher rate of postoperative prosthesis loosening for UCH while it may achieve shorter operation time and less intraoperative blood loss.

Several systematic reviews have been published in recent years trying to compare CH and UCH. Khan et al [7] performed a review involving 18 prospective and retrospective studies and claimed that in spite of its longer operation time and more intraoperative blood loss, CH possesses such advantages as better mobility, lower revision rate and less thigh pain without increasing postoperative complication and mortality rates at 1 month. A meta-analysis [8] involving 7 RCTs and 1 quasi-RCT revealed that CH was associated with significantly reduced pain at 3 months and during the next 1–2 years, and there was no significant difference between CH and UCH in terms of complications, reoperation rate, perioperative and postoperative mortality. The meta-analysis by Luo et al [9], which enrolled 8 RCTs (2 were indeed non-RCTs), demonstrated that there was no significant difference between CH and UCH regarding the mortality, reoperation rate and postoperative complications, while CH can reduce the risk of residual pain (RR = 0.69, 95% CI 0.53–0.90; P = 0.007; fixed-effects model) and achieve better functional recovery (a descriptive analysis). Azegami et al [10] performed a meta-analysis which pooled 8 RCTs and quasi-RCTs (the methodological quality of 2 trials ≤4 scores and the maximum quality score is 12 points) and reported that CH achieved better functional outcome and less residual pain. These studies, though compared many variables of the two techniques using meta-analysis, still need to be improved in three aspects. Firstly, predictable bias from quasi-RCTs or non-RCTs exists in all these studies. Secondly, complications of CH and UCH have not been stratified in the previous systematic reviews before comparison. Lastly, 2 latest RCTs, both of which compared the two techniques in treating elderly patients with femoral neck fractures and were published in 2012 have not been enrolled in any meta-analysis. We are therefore performing this meta-analysis with all the up-to-date RCTs to compare the effectiveness and safety of CH and UCH in treating femoral neck fractures in senior patients, in order to provide more accurate evidences for surgeons in making a clinical decision.

The specific questions that our study aims to answer include: (1) Does UH achieve better postoperative hip function and pain relief? (2) Is there any difference existing in the stratified postoperative complication rates between CH and UCH? (3) Do the mortality rates at different postoperative time points differ significantly between the two groups? (4) Which technique brings higher reoperation rate? (5) Is the operation time for CH significantly longer than that of UCH? (6) Is there any difference existing in the intraoperative blood loss between CH and UCH?

## Materials and Methods

### Search Strategies

A comprehensive search of unrestricted language literatures of all studies comparing CH with UCH was conducted through the online databases of PUBMED, EMBASE, Cochrane Library, CNKI (China National Knowledge Infrastructure) and VIP Database for Chinese Technical Periodicals from inception to December 2012. The following medical subject headings (MeSH) were searched: arthroplasty, hip fractures, femoral neck fracture. Hand-search of relevant trials, reviews and related articles were also performed.

### Inclusion Criteria/Exclusion Criteria

All randomized controlled trials comparing CH with UCH for femoral neck fractures in elderly patients were eligible. The participants should be over 70 years old and underwent primary hemiarthroplasty for unilateral femoral neck fractures. All kinds of prostheses were included without discrimination for this review. If there were more than one eligible publication from a same author, the one with either higher quality or the most recent publication date would be enrolled. All non-randomized trials and quasi-randomized trials were excluded.

### Outcomes of Interest

The primary outcomes include postoperative hip function, residual pain at 1 year, complications rates and mortality. Postoperative hip function outcomes at 2 months and 1 year were extracted and analyzed. We stratified complications into four categories. The first category includes such implanted-related complications as intraoperative and postoperative periprosthetic fractures, prosthesis loosening and dislocation. The second category includes cardiovascular and cerebrovascular complications such as intraoperative cardiac arrest, myocardial infarction, acute cardiac arrhythmia, intraoperative severe blood pressure reduction during preparation of femoral canal, cerebrovascular accidents, pulmonary embolism and deep venous thrombosis. The third category focuses on local complications including superficial or deep wound infection, wound hematoma, incision rupture and ectopic calcification. The last category includes general complications such as pneumonia, urinary tract infection, bedsore, gastrointestinal bleed, acute renal failure, etc. Mortality involves perioperative mortality, mortality at postoperative 1 month, 3 months and 1 year. The secondary outcomes consist of reoperation rate, operation time and intraoperative blood loss.

### Data Extraction

Two independent reviewers (Li T and Zhou L) extracted the data from all eligible randomized controlled trials. Any disagreement was resolved by discussion with a senior review author (Weng XS) and reasons for exclusion were recorded. If still more data was required for meta-analysis, communication through E-mail would be carried out with the primary authors for clarification.

### Quality Assessment

Two strategies were used to assess the methodological quality of eligibly studies. Firstly, all studies that met the criteria were assessed with the Jadad Scale Scoring System [11], in which the best study quality is scored 5 points. Studies with a score ≥3 points were considered as high quality research and were included in the this meta-analysis. Secondly, two independent reviewers (Bian YY and Zhuang QY) assess methodological quality of clinical trials using the Cochrane Collaboration recommendations. The trials were assessed in following aspects: random sequence generation, allocation concealment, blinding of outcome assessments, incomplete outcome data, selective reporting and other biases. An arbiter (Weng XS) was consulted to reconcile any disagreements.

### Evidence Grading

We graded the quality of evidences for our outcomes using the GRADE system (Grading of Recommendations Assessment, Development and Evaluation), and analyzed the data with GRADEprofiler software (version 3.6). Level of evidence strength were classified into: (1) High: further research is very unlikely to change our confidence in the estimate of effect. (2) Moderate: further research is likely to have an important impact on our confidence in the estimate of effect and may change the estimate. (3) Low: further research is very likely to have an important impact on our confidence in the estimate of effect and is likely to change the estimate. (4) Very low: we are very uncertain about the estimate.

### Statistical Analysis

For each included study, odds ratio (OR) and 95% confidence intervals (CI) were calculated for dichotomous outcomes, while weighted mean differences (WMD) and 95% CI were calculated for continuous outcomes. Statistical heterogeneity was assessed using the *I^2^* value and chi-squared test. A *p* value > 0.1 and an *I^2^* value ≤50% were considered as no statistical heterogeneity and an application of fixed-effects model was used to estimate the overall summary effect sizes. Otherwise, random-effects model was adopted and a subgroup analysis or sensitivity analysis would be carried out. All analyses were completed with Review Manager Software (RevMan 5.2) and *P* value <0.05 was considered as significant.

## Results

### Characteristics of Selected Studies

**Figure 1:**
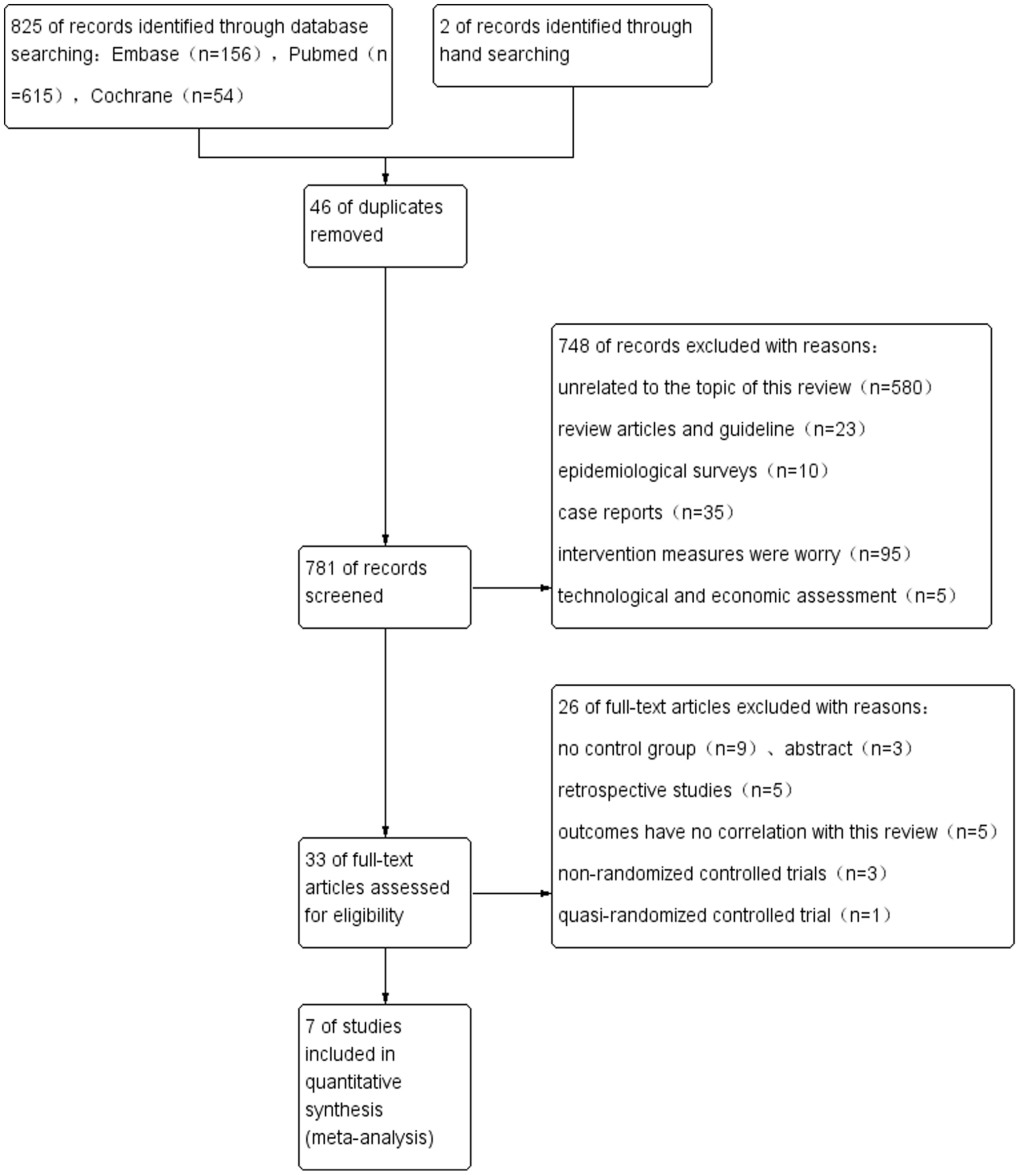
Flow diagram of searches.

The details of search and exclusion criteria are displayed in the flow diagram (Fig. 1). We finally identified 7 randomized controlled trials [12]–[18] associated with CH versus UCH. A total of 1,125 patients involving 1,125 hips ranging from 40 to 400 in each trial were included. All selected studies in our meta-analysis are in English and were published between 1977 and 2012. The follow-up period ranged from 12 months to 60 months. Each included trials present the baseline balance in age, sex, race, Charlson Comorbidity index and preoperative American Society of Anesthesiologists gradings. The characteristics of these studies are demonstrated in Table 1.

**Table 1:** Characteristics of included studies.

Included studies	Study period		Stage	Hips enrolled	Hips analyzed	Mean age (year)		Female (%)		Type of prosthesis	Follow-up		ITT	
						CH/UCH								
Taylor et al 2012 [18]		2006–2008	III,IV	160 (80/80)	160 (80/80)	85.3/85.1	69		Exeter/Alloclassic			24 m		Yes
DeAngelis et al 2012 [17]		2005–2008	III,IV	130 (66/64)	125 (64/61)	81.8/82.8	76.9		LD/Fx/ Beaded FullCoat			12 m		Yes
Parker et al 2010 [16]		2001–2006	III,IV	400 (200/200)	400 (200/200)	83/83	77		Thompson/Austin Moore			60 m		Yes
Figved et al 2009 [15]		2004–2006	III,IV	230 (115/115)	220 (112/108)	83.4/83.0	78/74		Spectron/HA-coated			24 m		No
Emery et al 1991 [14]		Unclear	III,IV	53 (27/26)	53 (27/26)	78/79.6	88.9/84.6		Thompson/Austin Moore			18 m		No
Sonne-Holm 1982 [13]		1979	Unclear	112 (57/55)	75 (35/40)	76	75		Austin Moore/Austin Moore			12 m		No
Sadr and Arden 1977 [12]		Unclear	III,IV	40 (20/20)	40 (20/20)	77/78.4	65/85		Thompson/Thompson			17 m		No

m: months.

ITT: intention to treatment.

### Risk of Bias

**Figure 2:**
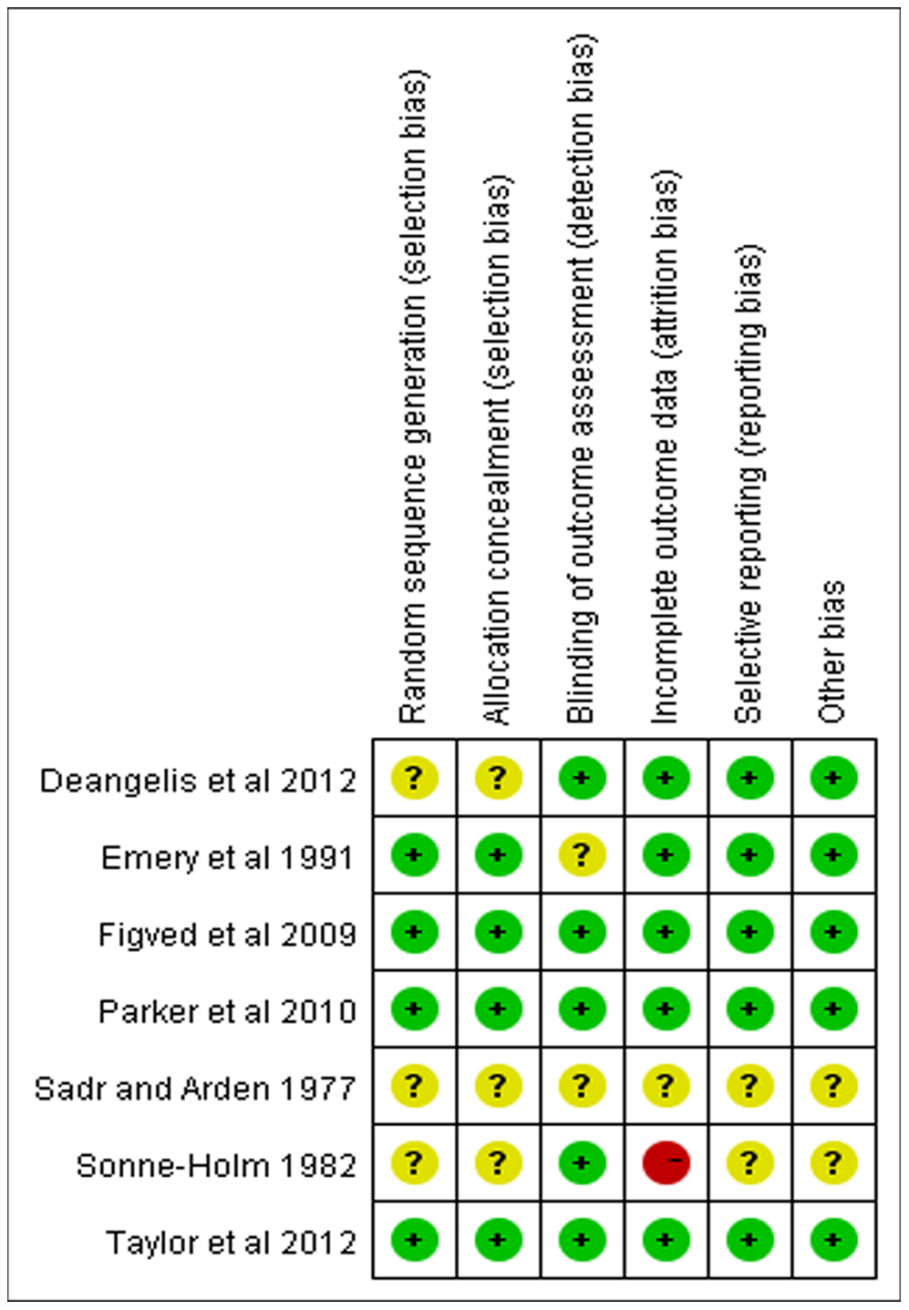
Quality assessment of risk of bias in included studies. “+”: low risk of bias, “?”: unclear risk of bias, “−”: high risk of bias.

**Figure 3:**
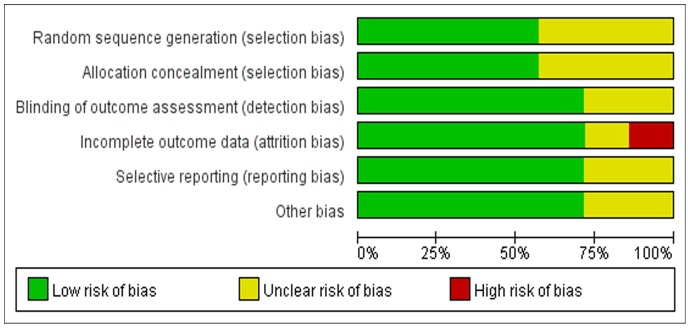
Summary of quality assessment of risk of bias.

In general, the methodological quality of all the trials were low in bias risk. The adequate randomization technique including a computer-generated number [15], [18] and a random numbered envelope [14], [16] was mentioned in 4 trials, and another 3 trials did not reported how the randomization was performed. Four trials mentioned allocation concealment [14]–[16], [18] while other 3 studies described unclearly. Outcome assessors were blinded in 5 studies [13], [15]–[18]. The detailed risk of bias about methodological quality of the included studies are elaborated and summarized respectively in Fig. 2 and Fig. 3.

### Meta-analysis Results

#### Postoperative hip function

**Figure 4:**
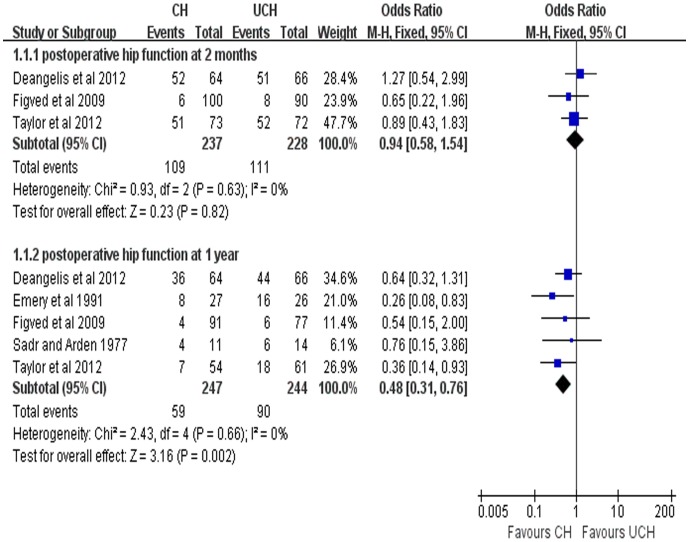
Forest plot of OR with confidence intervals for postoperative hip function.

Due to various outcome parameters adopted for the assessment of postoperative hip function, it was difficult to pool all the results. As the only comparable parameter, the number of patients requiring assistance with ambulation adopted by 5 trials was pooled. There was no significant difference between the two groups at 2 months (OR = 0.94, 95% CI, 0.58–1.54; P = 0.82). However, the odds ratio (OR) at 1 year was 0.48 (95% CI, 0.31–0.76; P = 0.002), indicating that postoperative hip function at 1 year in CH group was better than that in UCH group (Fig. 4). Moreover, a descriptive analysis was carried out, which indicated a tendency of better postoperative functional recovery for CH. 3 studies reported that better walking function recovery was achieved with both CH and UCH without no significant difference between the two techniques.

#### Residual pain

**Figure 5:**
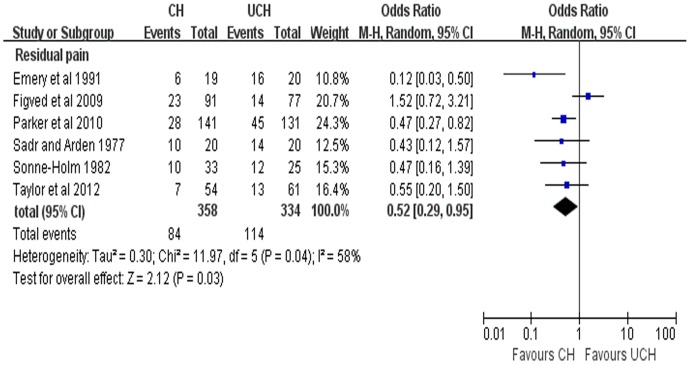
Forest plot of OR with confidence intervals for residual pain.

**Figure 6:**
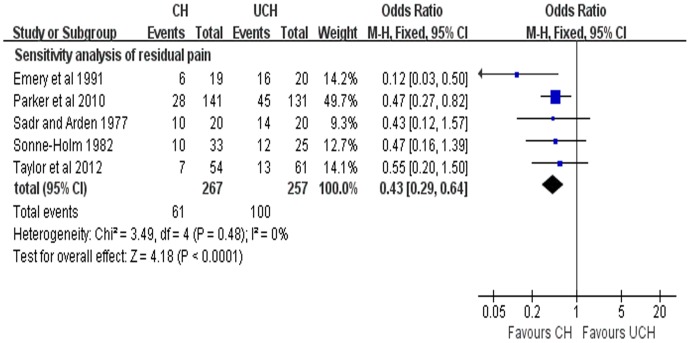
Forest plot of OR with confidence intervals for sensitivity analysis of residual pain.

6 studies reported the results of residual pain. Considering the high heterogeneity (P = 0.04, *I* ^2^ = 58%), random-effects model was adopted to pool the data and the results showed that CH can achieve less residual pain (OR = 0.52, 95% CI, 0.29–0.95; P = 0.03) (Fig. 5). A further sensitivity analysis was performed after 1 RCT [15] was excluded and the results were presented in Fig. 6. The sensitivity analysis revealed CH was associated less pain (OR = 0.43, 95%CI, 0.29–0.64; P<0.0001; fixed-effects model) with no heterogeneity (P = 0.48, *I^2^* = 0%) compared with UCH (Fig. 6).

#### Complications

**Figure 7:**
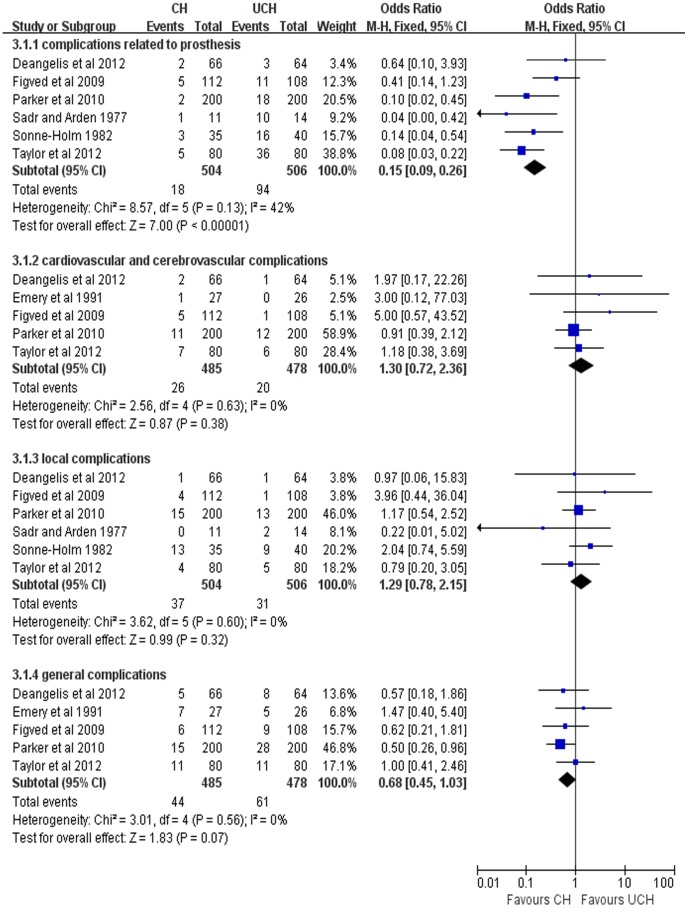
Forest plot of OR with confidence intervals for complications.

7 trials reported the complications in both CH and UCH groups. Odds ratio (OR) of implanted-related complications rates was 0.15, (95% CI, 0.09–0.26; P<0.00001) indicating that implanted-related complications rates in CH group were lower than that in UCH group. However, there was no significant difference between the two groups in cardiovascular and cerebrovascular complications (OR = 1.30, 95% CI, 0.72–2.36; P = 0.38), local complications (OR = 1.29, 95% CI, 0.78–2.15; P = 0.32) and general complications (OR = 0.68, 95% CI, 0.45–1.03; P = 0.07) (Fig. 7).

#### Mortality

**Figure 8:**
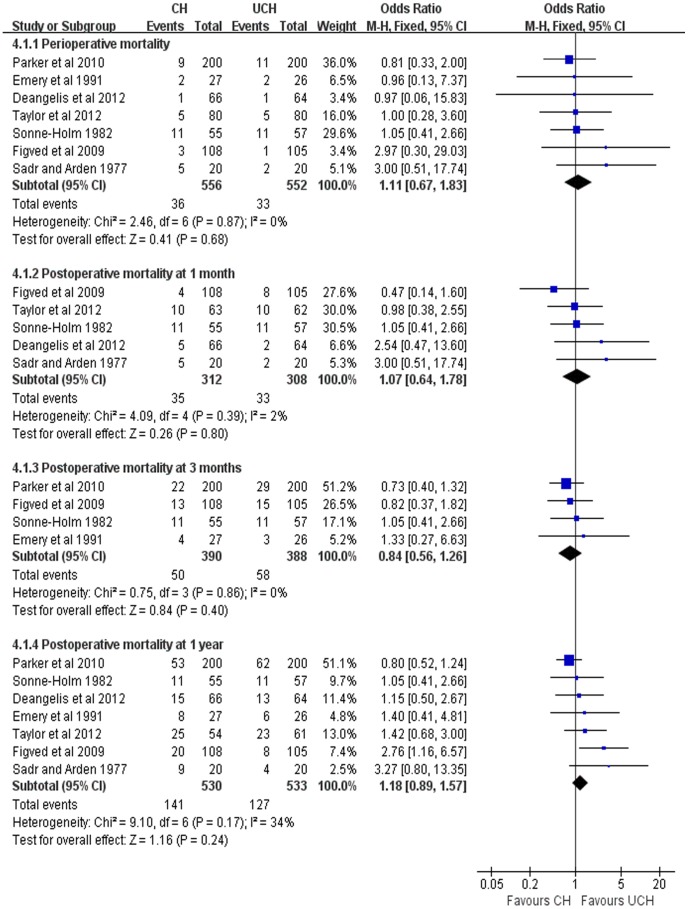
Forest plot of OR with confidence intervals for mortality.

All the 7 enrolled trials reported the mortality. Perioperative mortality in CH group did not differ from that in UCH group (OR = 1.11, 95% CI, 0.67–1.83; P = 0.68). Also, no significant difference was detected between the two groups for mortality at postoperative 1 month (OR = 1.07, 95% CI, 0.64–1.78; P = 0.80), 3 months (OR = 0.84, 95% CI, 0.56–1.26; P = 0.40) and 12 months (OR = 1.18, 95% CI, 0.89–1.57; P = 0.24) (Fig. 8).

#### Reoperation rate

**Figure 9:**
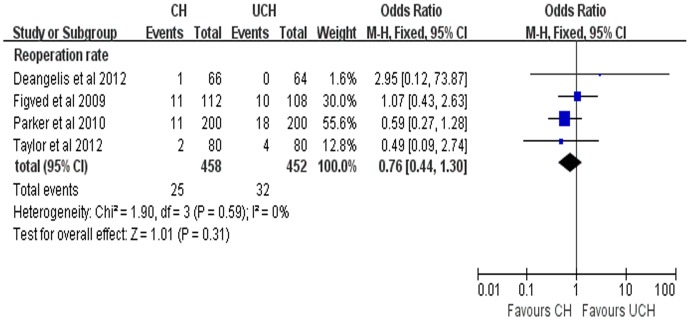
Forest plot of OR with confidence intervals for reoperation rate.

4 included studies reported reoperation rate for CH and UCH. The pooled results showed that there was no significant difference between the compared groups in the reoperation (OR = 0.76, 95% CI, 0.44–1.30; P = 0.31) (Fig. 9).

#### Operation time

**Figure 10:**
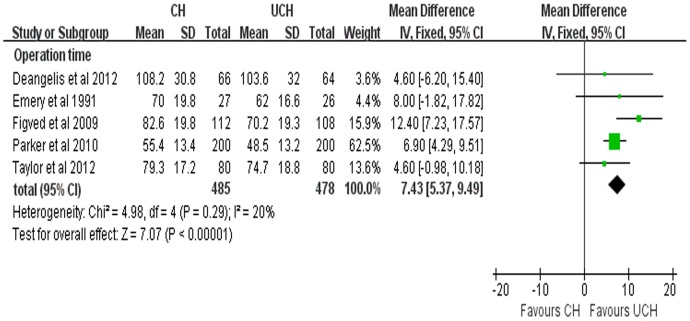
Forest plot of WMD with confidence intervals for operation time.

This parameter was measured in 5 enrolled studies. The weighted mean differences (WMD) of operation time was 7.43 min (95% CI, 5.37–9.49 min; P<0.00001), indicating that a shorter operation time was achieved in CH group than that in UCH group (Fig. 10).

#### Intraoperative blood loss

**Figure 11:**
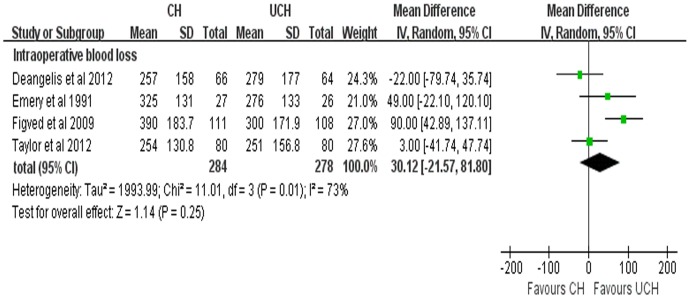
Forest plot of WMD with confidence intervals for intraoperative blood loss.

**Figure 12:**
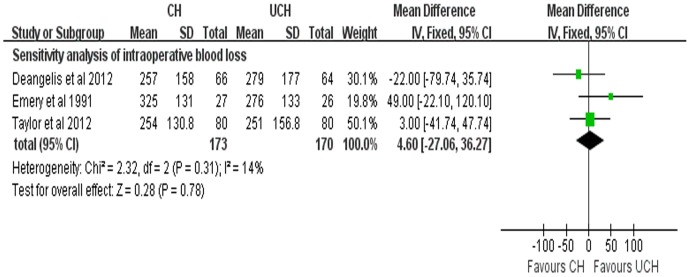
Forest plot of WMD with confidence intervals for sensitivity analysis of intraoperative blood loss.

4 studies reported the results of intraoperative blood loss. Due to the high heterogeneity (P = 0.01, *I* ^2^ = 73%), random-effects model was adopted to pool the data. There was no significant difference between the two groups in the intraoperative blood loss (WMD = 30.12 ml, 95% CI, −21.57–81.80 ml; P = 0.25) (Fig. 11). A further sensitivity analysis was performed after 1 RCT [15] was excluded and the results were presented in Fig. 12. The sensitivity analysis indicated that no significant difference (WMD = 4.60 ml, 95%CI, −27.06–36.27 ml; P = 0.78; fixed-effects model) with low heterogeneity (P = 0.31, *I^2^* = 14%) between the two groups, which was consistent with our previous analysis.

#### GRADE system assessment

**Figure 13:**
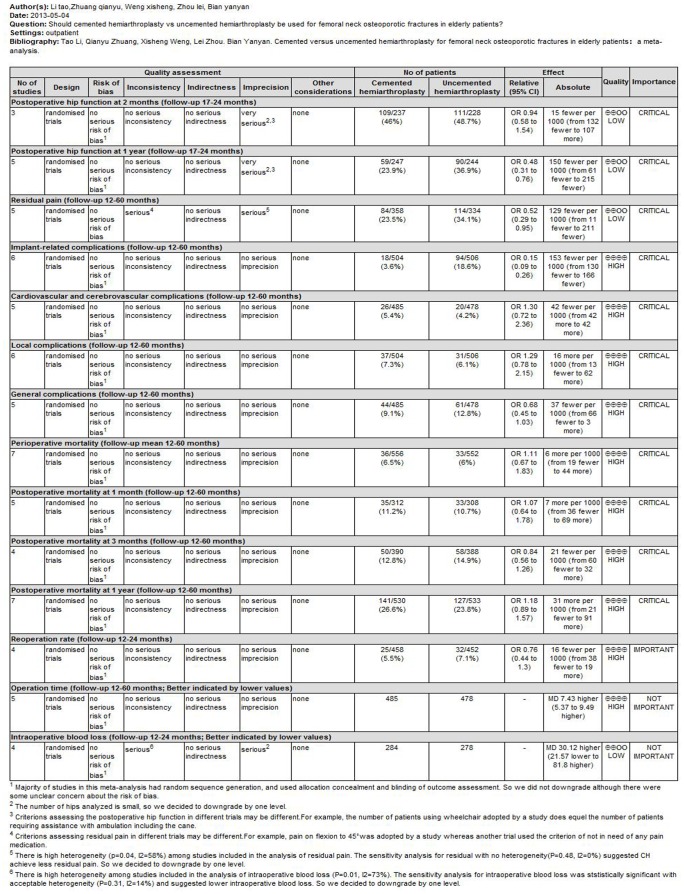
Summary of finding for the comparision and the quality of evidence.

A summary of our results and the strength of evidence assessed through GRADE system were displayed in Fig. 13. The strength of evidence was high for complications, mortality, reoperation rate and operation time, but low for postoperative hip function recovery, residual pain, and intraoperative blood loss.

## Discussion

With the trend of global aging, femoral neck fracture has become an increasingly serious problem for senior patients. Hemiarthroplasty, as an effective treatment, can help resume the walking ability as soon as possible, thereby reduce the risk of respiratory infection and urinary tract infection. However, there has been controversy regarding the use of cement for a long time. Some surgeons prefer to apply the UCH technique since it can reduce operation time, intraoperative blood loss and perioperative cardiovascular complications, while others believe that the CH technique can achieve better postoperative hip function recovery and less prosthesis loosening. We therefore performed this meta-analysis with all the up-to-date RCTs concerning the comparison of CH and UCH techniques for femoral neck fractures.

In this study, we pooled the number of patients requiring assistance with ambulation from each trial, which was the only common parameter from the enrolled trials in assessing the postoperative hip function. Although we could not demonstrate a statistically significant difference between the 2 groups at 2 months, there was a trend towards better postoperative functional recovery for CH at this time point. The postoperative hip function at 1 year was better in CH group than that in UCH group, indicating that with the time passing after the operation, CH technique can bring better joint function recovery, which is consistent with previous studies[7], [9]–[10]. In a retrospective study involving 447 patients with 451 displaced fractures of femoral neck treated by Bateman bipolar hemiarthroplasty, Lo et al [19] found that the cemented prostheses brought better functional results in the early stage. Khan’s study [20] using validated scoring systems for pain and functional ability assessment demonstrated that there was significant deterioration in pain (P = 0.003), walking ability (P = 0.002), and daily activities (P = 0.009) in the UCH group during the follow-up of 32–36 months. Other researches[21]–[22] suggested that there was no clinically or statistically significant difference in the postoperative hip function recovery. In spite of an obvious tendency for CH in postoperative function recovery, it was difficult to pool and compare other parameters due to the inconsistency of outcome parameters applied. Further researches with large samples and standardized hip function scoring systems are warranted to confirm these findings and elucidate the potential advantages of CH in postoperative hip function recovery.

With regards to the residual pain, 6 included studies reported related results and the pooled results showed that CH have less residual pain compared with UCH with high heterogeneity (P = 0.04, *I* ^2^ = 58%). It worth noting that one enrolled trial [15] adopted hydroxylapatite coated implant while other four trials used non-hydroxylapatite coated prostheses including Austin Moore prostheses and Alloclassic implants. As an earlier randomized trial reported, better pain relief was achieved with uncemented hydroxylapatite coated implant than with the Austin Moore prosthesis [23]. We therefore speculated that the high heterogeneity in our analysis result from the different types of uncemented prostheses used among the trials we enrolled. In order to further explore the source of heterogeneity, a further sensitivity analysis was performed with this trial [15] being excluded and the pooled results with no heterogeneity (P = 0.48, *I* ^2^ = 0%) showed that CH was associated with less residual pain, which was consistent with our previous results. Several non-RCT studies[19]–[20], [24] also support our findings, reporting that CH led to less residual pain than UCH with significant difference.

We also found a significantly higher implant-related complications associated with CH than UCH. Our result was in agreement with a previous study [25] which indicated that UCH led to more intraoperative and postoperative periprosthetic fractures, prosthesis loosening, dislocation. Therefore surgeons should pay more attention to these possible complications prior to surgery. However, no significant difference was found between the two groups in cardiovascular and cerebrovascular complications, although previous studies[3]–[6], [26]–[27] revealed that the cement insertion might increase the danger of transient hypotension and hypoxaemia, pulmonary embolism, and cardiovascular system accidents. Furthermore, there was no significant difference between the two groups in local complications and general complications, indicating that cement play little, if not none, role in local and general complications. Interestingly, a recent large scale retrospective study [25] comparing CH with UCH involving 60,848 patients showed that cementless implants were related with significantly higher rates of myocardial infarction (2.86% versus 2.46%, OR = 1.17, 95%CI, 1.07–1.28) and lower respiratory tract infection (9.21% versus 7.26%, OR = 1.15, 95% (1.09–1.22), p<0.001) within 30 days compared with cemented implants. Certainly, high quality evidences with well-designed RCTs are still required.

Previous studies[26]–[27] showed that the cement may play an important role in mortality increase due to its possible risk of inducing cerebrovascular complications and cardiovascular events. Nevertheless, our study found no significant difference between CH group and UCH group in perioperative mortality and mortality at postoperative 1 month, 3 months, and 1 year, indicating that the use of cement does not increase the aforementioned risks. Only one study [15] reported that one patient experienced severe reduction of blood pressure during the cementing procedure and died within 24 hours of a myocardial infarction, and another patient developed intraoperative cardiac arrest during wound closure. Other studies [22], [24] demonstrated that the mortality rate at 12 months of follow-up was similar between the two groups. Besides, old age, deteriorated preoperative cardiopulmonary function and physical reserve have been regarded as risk factors recently[28]–[29].

In addition, there was a tendency of higher reoperation rate in UCH group although no significant difference was found between the two groups in the meta-analysis. The aforementioned national retrospective study [25] involving 60,848 matched patients supported our findings by demonstrating that revision rates in UCH group were higher than that in CH group at 18 months (1.66% versus 0.67%, OR = 2.90, 95%CI 2.50–3.37, p<0.001) and 4 years (2.56% versus 1.39%, OR = 2.22, 95%CI 1.84–2.70, p<0.001).

Our meta-analysis demonstrated that CH was related with significantly prolonged operation time, which was consistent with the studies by Khan [7] and Azegami [10]. It may result from the process of cement insertion and the waiting time for the solidification of cement. As for intraoperative blood loss, the pooled results with high heterogeneity (P = 0.01, *I* ^2^ = 73%) showed that there was no significant difference between CH group and UCH group. We speculated that the inconsistency of types of prostheses used in these studies may be the possible explanation of the high heterogeneity. As we discussed above, one trial [15] adopted hydroxylapatite coated implant while other three trials used non-hydroxylapatite coated prostheses, which was regarded as the source of heterogeneity. In order to decrease the heterogeneity to an acceptable level, a further sensitivity analysis was conducted with this trial [15] being excluded and the pooled results with low heterogeneity (P = 0.31, *I* ^2^ = 14%) were in agreement with our previous analysis.

The Grading of Recommendations Assessment, Development and Evaluation (GRADE) recommended by The Cochrane Collaboration provides a system for rating quality of evidence and strength of recommendations that is explicit, comprehensive, transparent, and pragmatic and is increasingly being adopted by organizations worldwide. In this meta-analysis, we adopted the GRADE system to evaluate our results. The quality of evidence of most outcomes in our study was high. However, evidence strength for postoperative hip function, residual pain, and intraoperative blood loss were graded as low due to following reasons: (1) The hip sample size was relatively small in postoperative hip function, residual pain, and intraoperative blood loss analysis. (2) Criterions assessing the postoperative hip function in different trials may be different. (3) Criterions assessing residual pain in different trials may be different. For example, pain on flexion to 45°was adopted by a study whereas another trial adopted free of pain medication as the criterion. (4) There is high heterogeneity among studies included in the analysis of residual pain (P = 0.04, *I^2^* = 58%) and intraoperative blood loss (P = 0.01, *I^2^* = 73%).

Compared with previous systematic reviews, there are several improvements in this meta-analysis. Firstly, this meta-analysis adopted more strict inclusion criteria. Quasi-RCT and non-RCTs were strictly excluded in this study in order to guarantee the reliability of results. Secondly, two strategies were used to assess the methodological quality of the included studies. All the included studies were of highly qualified methodology according to the quality assessment system, which contributes to the strength of conclusions drawn from the meta-analysis. Thirdly, complications were further stratified into implanted-related complications, cardiovascular and cerebrovascular complications, local complications and general complication, reducing the potential bias risk from pooling all kinds of complications. Fourthly, we pooled the data of one comparable parameter regarding postoperative hip function to reduce the bias of the descriptive analysis. Lastly, GRADE system was adopted for the assessment of the quality of evidences so as to better guide the clinical practice better.

Despite these advantages, some limitations are still recognized. Firstly, the number of trials included in the study is still relatively small and it is therefore difficult for us to conduct funnel plots to assess the publication bias. Secondly, various types of prostheses involved in this study may bring related bias. Thirdly, since the outcome parameters in different trials were different, it is impossible to pool all kinds of parameters regarding hip function. Instead, only one parameter was analyzed in our study. Lastly, only short and middle term follow-up data are available and long term follow-up results still need disclosing in the future.

In summary, our study, as the first meta-analysis composed only of RCTs, compared cemented and uncemented hemiarthroplasty for femoral neck fractures in elderly patients. Our results suggested that CH technique, compared with UCH, is related with better hip function recovery, lower residual pain, less implant-related complications. There was no significant overall difference in mortality rate, cardiovascular and cerebrovascular complications, general complications, local complications and reoperation rate. Multicenter randomized controlled trials with large samples are still needed in the future to verify our results.

## Supporting Information

Checklist S1 **PRISMA 2009 Checklist.**
(DOC)
